# Lactic Acid Fermentation as a Pre-Treatment Process for Faba Bean Flour and Its Effect on Textural, Structural and Nutritional Properties of Protein-Enriched Gluten-Free Faba Bean Breads

**DOI:** 10.3390/foods8100431

**Published:** 2019-09-21

**Authors:** Nesli Sozer, Leena Melama, Selim Silbir, Carlo G. Rizzello, Laura Flander, Kaisa Poutanen

**Affiliations:** 1 VTT Technical Research Centre of Finland Ltd., Tietotie 2, FI02044 Espoo, Finland; leena.melama@unilever.com (L.M.); selim.silbir@ege.edu.tr (S.S.); laura.flander@perheleipurit.fi (L.F.); kaisa.poutanen@vtt.fi (K.P.); 2 Food Engineering Department, Ege University, Izmir 35040, Turkey; 3 Department of Soil, Plant, and Food Science, University of Bari, 70121 Bari, Italy; carlogiuseppe.rizzello@uniba.it; 4 Bakery Primula Ltd., 04440 Järvenpää, Finland

**Keywords:** gluten-free, legumes, faba beans, fermentation, textural properties, nutritional properties

## Abstract

Lactic acid fermentation could be used as a potential modification tool for faba bean flour to enable its incorporation in boosting the nutritional profile of gluten-free breads. Gluten-free breads made with fermented or unfermented faba bean flours were compared with commercial soy flour. The amounts of faba- and soy-bean flours were adjusted to obtain the same protein content in bread (16%). Both fermented and unfermented faba bean flour resulted in larger bread volume (2.1 mL/g and 2.4 mL/g, respectively) compared to bread made with soybean flour (1.5 mL/g). Breads made with unfermented and fermented faba flour had higher porosity (82% and 72%, respectively) than bread with soy flour (61%). The faba breads also were softer than the soy bread. Fermentation of faba flour prior to bread making significantly increased crumb hardness (584 vs. 817 g). Fermentation increased in vitro protein digestibility (72.3% vs. 64.8%). Essential Amino Acid and Biological Value indexes were significantly higher for breads containing fermented faba flour compared to breads made with unfermented faba and soy flour. The Protein Efficiency Ratio and Nutritional Index increased by fermentation from 33 to 36 and 1.6 to 2.7, respectively. Pre-fermentation of faba bean flour improved the nutritional properties of high-protein, gluten-free faba bread. A sensory panel indicated that fermentation did not affect the crumbliness, evenness of pore size and springiness of breadcrumb.

## 1. Introduction

Faba bean (*Vicia faba*) is an ancient crop, and together with pea, is one of the most important legume crops in Europe. Faba bean seeds, used in both human and animal nutrition, are rich in essential amino acids: isoleucine, leucine, lysine, phenylalanine, threonine and valine [1,2], and when combined with cereal ingredients (rich in sulphur-containing amino acids) make a plant protein source with well-balanced amino acid composition [3]. Faba bean is also rich in dietary fibre, minerals, phenolics, and non-nutrient secondary metabolites recognized as beneficial in human health [4]. Faba bean storage proteins have been found to lower serum glucose, insulin, total and low-density lipoprotein cholesterol in young men with hypercholesterolemia, whereas l-3,4-dihydroxyphenylalanine (l-DOPA), contained at high levels in seeds, has been reported as effective in the treatment of Parkinson’s disease [5,6]. Despite the nutritional and functional properties, faba bean use is limited by the presence of antinutritional factors (ANF), which can interfere with digestion, sometimes causing pathologic conditions. The main ANF are α-galactosides (e.g., raffinose, verbascose, stachyose), condensed tannins, protease inhibitors, phytic acid and vicine and convicine [4,6,7]. The pyrimidine glycosides, vicine and convicine, responsible for causing favism in susceptible individuals (suffering from glucose 6 phosphate dehydrogenase deficiency) [6], are synthesized by the plant as a defence mechanism against fungi and insects [8]. In addition, lectins, found in most legumes, can cause gastrointestinal tract distress [9].

Some of these ANF are heat-labile (e.g., protease inhibitors and lectins) and thus thermal treatments would remove the negative effect during consumption. Others (e.g., phytic acid, raffinose, tannins, saponins) are heat stable [4]. Processing, such as dehulling [10,11], boiling and roasting [12], and germination [13], have been suggested to reduce the ANF content of legumes. Nonetheless, fermentation through lactic acid bacteria (LAB) has proven to be effective in ANF reduction [6,14,15,16,17]. Overall, LAB fermentation is a traditional food processing method with many applications, including baked products [18]. It can provide improved sensory, technological and nutritional properties as well as longer shelf life [18]. Recently, fermentation has been reported as being responsible for the enhancement of the overall nutritional and functional quality of faba bean through the increase of the protein digestibility, the release of bioactive peptides and free aminoacids, the decrease of the glycemic index, the increase of mineral bioavailability and antioxidant activity, and by producing γ-aminobutyric acid (GABA), a non-protein amino acid with health promoting effects [6,14,15,16,17]. Moreover, the degradation of the pyrimidine glycosides, vicine and convicine, was complete after 48 h of fermentation with a LAB strain selected for the high β-glucosidase activity, and the aglycone derivatives (also toxic) were not detectable. Ex-vivo assays on human blood also confirmed the lack of toxicity of fermented faba bean flour [18].

Like other legume crops, faba bean is gluten-free and could thus be utilized for gluten-sensitive and celiac people. The prevalence of celiac disease has become more and more common in recent years, therefore, the need for tasty and nutritious gluten-free foods is increasing. The structure and texture of gluten-free foods pose challenges for baking technology. Pulses such as chickpea, pea or soybeans have been used in gluten-free bread baking either in the form of flour or protein concentrate supplemented to a starch base [19,20]. Chickpea protein has good emulsifying properties and its supplementation to corn starch (8% of flour mix) improved bread volume, crust/crumb colour and crumb hardness without the need of shortening or emulsifier compared to breads made with cornstarch [19]. Chickpea flour-based gluten-free bread had the best physico-chemical and sensory characteristics compared to bread made with soy or pea protein isolate with equal protein content [20].

We have previously shown that fermentation of faba bean flour by *Lactobacillus plantarum* decreased ANFs, increased the amount of free essential amino acids, improved in vitro protein digestibility and lowered the starch hydrolysis index [13]. After fermentation of faba flour, vicine and convicine contents reduced from 11.46 ± 0.22 to 0.67 ± 0.01 (mg/g dm) and 6.24 ± 0.14 to 0.56 ± 0.01 (mg/g dm), respectively [13]. Trypsin inhibitor (TI) activity dropped from 2.09 ± 0.09 to 0.91 ± 0.04 (TI unit/mg dm), whereas the concentration of condensed tannins halved from 27.10 ± 1.30 to 13.71 ± 0.79 (eq catechin/100 g) by fermentation [13]. The objective of the current work was to evaluate the applicability of fermented faba bean flour for protein enrichment of gluten-free bread especially with respect to structural, textural and nutritional properties.

## 2. Materials and Methods

### 2.1. Materials

Faba beans (*Vicia faba* cv Kontu), which were used for the production of faba bean flour, were provided by the University of Helsinki, Finland. The beans were dehulled by using an ultra-fine friction grinder (Supermasscolloider MKZA10-15, Masuko Sangyo Co. Ltd., Kawaguchi, Japan) operated with a 0.5 mm gap, and the hulls were aspirated in a spray drier (Mobile minor, GEA Niro A/S, Soeborg Denmark). Dehulled beans were milled as described in Reference [13] by using a cutting mill (Retsch GmbH, Haan, Germany) to obtain the faba bean flour. Commercial soy flour (native, not heat-treated) (Soyappetit, Helsinki, Finland) (protein 40.3%, fat 21.6%, total dietary fibre 14.2%, total carbohydrates including sugar, 13.3%), corn starch (Maizena, Unilever, Denmark) (starch 86%, total dietary fibre 1%, protein and sugar <0.5%), sugar (Dan Sucker, Suomen sokeri Oy, Kantvik, Finland), baking powder (Meira, Helsinki, Finland), shortening (Sunnuntai, Raisio, Finland), baker’s yeast (Suomen hiiva Oy, Lahti, Finland), and salt (JOZO salt, Mariager, Denmark) were obtained from a local store. In addition, emulsifier (Panodan A2020, Dupont, Wilmington, DE, USA) and xanthan gum (Carl Roth GmbH, Karlsruhe, Germany) were purchased.

### 2.2. Fermentation of Faba Bean Flour with Lactic Acid Bacteria

#### 2.2.1. Microorganisms and Culture Condition

*Lactobacillus plantarum* VTT E-133328, at VTT Culture Collection (Espoo, Finland) was routinely propagated at 30 °C in MRS broth (Oxoid, Basingstoke, Hampshire, England). When used for fermentation, lactic acid bacterial cells were cultivated until the late exponential phase of growth was reached (ca. 10 h), washed twice in 50 mM phosphate buffer, pH 7.0, and suspended in the tap water used for fermentation.

#### 2.2.2. Fermentation Procedure

Faba bean flour was mixed with water to prepare dough in a ratio of 50:50. Fermentation with the lactic acid bacteria (initial cell density of ca. 10^7^ CFU/g of dough) was carried out at 30 °C for 48 h as described in Reference [13]. After fermentation, the pH of faba flour was 4.1 and adjusted to higher pH levels around 5.5 by using sodiumbicarbonate to avoid excessive sour taste in the breads. The dough was later freeze-dried (Christ Alpha 2-4 B. Braun, Bioteck International, Osterode am Harz, Germany) and milled in an ultra-centrifugal mill ZM 200 (Retsch GmbH, Haan, Germany) using a 0.5 mm screen and 8000 rpm speed.

The pH drop during fermentation was measured by a TitroLine autotitrator (Alpha 471217, Schott, Mainz, Germany) suspending and aliquot of 10 g of samples in 100 mL distilled water.

Total titratable acidity (TTA) was determined with the TitroLine Alpha autotitrator on 10 g dough homogenized with 90 mL of distilled water and expressed as the amount (ml) of NaOH 0.1 M to reach pH of 8.5.

#### 2.2.3. Pasting Properties by Rapid Visco Analyzer

The Rapid ViscoTM Analyser (RVA) (Newport Scientific Pty Ltd., Warriewood, Australia) was used to determine the pasting properties of the faba bean flours. Pasting properties were determined following the standard Newport Scientific Method 1 (STD1). The heating cycle was 50–95 °C in 282 s, holding at 95 °C for 150 s, and then cooling down to 50 °C in 228 s and holding at 50 °C for 120 s. Each cycle was initiated by a 10 s mixing at 960 rpm paddle speed and 160 rpm paddle speed was used for the rest of the test. The RVA studies were carried out using 3.5 g of dried sample and 25 mL deionized water in an aluminium canister. The parameters recorded were peak, final, breakdown and setback viscosities together with pasting temperature. Flour samples were run in triplicate.

#### 2.2.4. Baking of Gluten-Free Breads

Faba bean flour and fermented faba bean flour (freeze-dried) were applied in gluten-free bread baking and commercial soy flour was used for control breads. The amounts of bean flours were adjusted to obtain the same final protein content (16%) which would enable the “protein-rich” nutritional claim. Bread S37 was soy bread with 37% soy flour and 63% corn starch, bread F50 was faba bread with 50% faba flour and 50% corn starch, and bread FF50 was faba bread with 50% fermented faba flour and 50% corn starch. The proportion of the rest of the ingredients were fixed for each bread recipe and were as follows: sugar 5.7%, baking powder 2.4%, shortening 4.9%, baker’s compressed fresh yeast 4.9%, salt 1.7%, xanthan gum 2%, emulsifier 0.6%, water 102.7%. All the percentages were based on baker’s percentages. The recipe, mixing, proofing time and baking conditions were optimized in a preliminary work which is not shown here. The dry ingredients were first mixed together and then shortening, yeast and water were added. The temperature of the water was adjusted so that the final dough temperature after mixing was 26 ± 1 °C. Kneading was done for 4 minutes at high speed (level 2/3) with a planetary mixer (Ningbo Sybo Machinery Co., B30CT, Ningbo, China). Small tins (9 × 5 × 6 cm) were filled with 160 g of batter and then proofed for 45 min at 28 °C and 85% relative humidity The breads were baked in a rack oven (Sveba Dahlen, Fristad, Sweden) at 180 °C for 17 min. Baking was conducted twice for each bread recipe.

### 2.3. Texture Profile Analysis

The texture profile analysis of the crumb was performed after 1 h and 24 h of baking. Two slices were taken from the centre of each loaf. Uniform slices of 2.5 cm thickness were obtained with a cutter and the analysis was carried out with a TA-XT Plus texture analyser (Stable micro systems, Godalming, UK), equipped with a 25 kg load cell and a 25 mm diameter aluminium cylindrical probe. The pre-test and test speeds were identical with a value of 1.7 mm/sand post-test speed was 10 mm/s. A strain value of 40% strain with 5 s pause time between compression cycles was applied. The results are an average of ten replicates.

### 2.4. Volume Analysis

The specific volume of the bread loaves was measured based on the infrared light scanning method (Bread Vol Scan, Pregesbauer, Germany). Six loaves of each bread type were measured, and the average value was calculated.

### 2.5. Sensory Analysis

A trained descriptive sensory panel (*n* = 5) evaluated the characteristics of the breads based on a previously published protocol [21]. The panel consisted of two males and three females with an age range of 27–50 years. Attribute intensities were rated on 5-unit, verbally anchored intensity scales. Altogether, three attribute categories were selected to describe the texture and flavour of the breads. These were: appearance (Evenness of bread on a scale in which 1 = not at all even and 5 = very even, Shape of bread on a scale in which 1 = very asymmetric and 5 = very symmetric, Intensity of bread colour on a scale in which 1 = very light and 5 = very dark), Texture of the crumb (Crumbliness on a scale in which 1 = not at all crumbly and 5 = very crumbly, Evenness of the pore size on a scale in which 1 = very uneven (big pore >20 mm) and 5 = very even, Softness of the crumb on a scale in which 1 = firm and 5 = very soft, Springiness of the crumb on a scale in which 1 = not at all springy (the slice is broken when bended slightly) and 5 = very springy, Toughness on a scale in which 1 = not at all tough (needs only 2 bites before broken down) and 5 = very tough), Crumb flavour and colour (Intensity of the colour on a scale in which 1 = very light and 5 = very dark, Intensity of the flavour on a scale in which 1 = flavourless and 5 = very intense flavour).

### 2.6. Microstructural Analyses

#### 2.6.1. X-ray Microtomography

Samples for X-ray microtomography (XMT) were made by cutting 1 cubic cm bread samples at 3 different locations from the centre of each bread recipe. Samples were scanned using a desktop XMT system (Model 1172, SkyScan, Aartselaar, Belgium) consisting of an X-ray tube, an X-ray detector and a charge-coupled devices (CCD) camera. The X-ray tube was operated at a voltage of 40 kV/250 μA to obtain optimum contrast between void (air cells) and matter (cell walls) according to a modified method [22]. A 12-bit cooled CCD camera (524 × 1984 pixels) was used to collect the X-ray data. Samples were rotated by a total of 180° during the scanning process with a pixel size of 12.85 μm to obtain optimum resolution, resulting in a total scanning time of 33 min. The initial X-ray radiographs or raw images were obtained at every 0.7° of rotation. Samples were scanned in triplicate. After scanning, radiographs were loaded into NRecon reconstruction software (v. 1.6.6, Micro Photonics, Allentown, PA, USA). The software combines the images graphically into a three-dimensional (3D) object from which 2D cross-sectional images can be taken. Before the reconstruction, the cross-sectionrotation feature was used to rotate the sample cross-sections, making them parallel to the view window. Beam hardening correction and ring artefact corrections were set to 10% and 40% respectively, in order to reduce the number of artefacts. Cell walls of the solid matrix appear grey, whereas air cells appear black. The reconstructed 2D slices were then loaded into CTAn software (v. 1.12, Skyscan, Kontich, Belgium) to obtain the parameters of porosity (%), cell wall thickness (t), and cell diameter (D).

#### 2.6.2. Light Microscopy

Bread samples were first embedded in 2% (*w*/*v*) agar and fixed in 1% (*v*/*v*) glutaraldehyde in 0.1 M Na-K phosphate buffer (pH 7.0), dehydrated in a graded ethanol series, and embedded in hydroxyethyl methylacrylate resin (Leica Historesin embedding kit, Leica Microsystems, Heidelberg, Germany). Polymerized samples were sectioned (2 µm sections) in a rotary microtome HM 355S (Microm Laborgeräte GmbH, Walldorf, Germany) using a tungsten carbon knife and stained after transferring onto glass slides. Staining of protein and starch in 2 mm thick sections were done with Light Green and Lugol’s iodine respectively, as described in Reference [13]. The stained sections were examined with an Olympus BX-50 microscope (Olympus Corp., Tokyo, Japan). Micrographs were obtained using a PCO SensiCam CCD colour camera (PCO AG, Kelheim, Germany) and the Cell^P imaging software (Olympus, Tokyo, Japan).

### 2.7. Nutritional Properties 

In vitro starch digestibility was measured based on Reference [23] and represented as hydrolysis index (HI) which refers to the amount of maltose per 1 g of soluble starch as compared to wheat bread. Triplicate sample suspensions containing 1 g of starch in 0.0 5M sodium potassium phosphate buffer (pH 6.9) were placed in water bath (37 °C) and the pH was adjusted to 6.9 with 1M NaOH and 1N HCl. Pancreatic amylase (110 U) was added to the suspensions. Sample aliquots were removed before the enzyme addition and after 30, 60, 120 and 180 min and placed in a boiling water bath for 5 min followed by cooling. Samples were later on analysed for reducing sugar content and absorbance was measured at 540 nm against the reagent blank. The calibration curve was made with maltose. White wheat bread (Isopaahto, Vaasan and Vaasan, Espoo, Finland) was used as control sample.

In vitro protein digestibility was measured based on Akeson and Stahman [24]. One gram of each sample was incubated with 1.5 mg of pepsin in 15 mL of 0.1M HCl at 37 °C for 3 h. After neutralization with 2 M NaOH and addition of 4 mg of pancreatinin 7.5 mL of phosphate buffer (pH 8.0), 1 mL of toluene was added to prevent microbial growth, and the solution was incubated for 24 h at 37 °C. After 24 h, the enzyme was inactivated by the addition of 10 mL of trichloroacetic acid (20%, *w*/*v*), and the undigested protein was precipitated. The volume was made up to 100 mL with distilled water and the mixture centrifuged at 5000 rpm for 20 min. The precipitate was subjected to protein extraction according to Weiss et al. [25]. The concentration of protein in the supernatant and the precipitate was determined by the Bradford method [26]. The in vitro protein digestibility was expressed as the percentage of the total protein, which was solubilized after enzyme hydrolysis.

The supernatant, which contained the digested protein, was freeze-dried and used for further analyses. The method of AOAC 982.30a [27], modified as previously reported by Curiel et al. [28], was used to determine the amino acid profile of the digested protein fraction. Amino acids were analysed by a Biochrom 30 series Amino Acid Analyser [28], with the exception of tryptophan, estimated by the method of Pintér-Szakács and Molnán-Perl [29].

Chemical Score (CS) estimates the amount of protein required to provide the minimal essential amino acid (EAA) pattern, which is present in the reference protein (hen’s egg). It was calculated using the equation of Block and Mitchel [30]. The sequence of limiting essential amino acids corresponds to the list of EAA, having the lowest chemical score [30]. The protein score indicates the chemical score of the most limiting EAA that is present in the test protein [30].

Essential Amino Acids Index (EAAI) estimates the quality of the test protein, using its EAA content as the criterion. EAAI was calculated according to the procedure of Oser [31], according to the following equation:

(1) $$EAAI=\sqrt[{n}]{\frac{\left(EAA_{1}\times 100\right)\left(EAA_{2}\times 100\right)\left(\ldots \right)\left(EAA_{n}\times 100\right)\left[sample\right]}{\left(EAA_{1}\times 100\right)\left(EAA_{2}\times 100\right)\left(\ldots \right)\left(EAA_{n}\times 100\right)\left[reference\right]}}.$$

The Biological Value (BV) indicates the utilizable fraction of the test protein. BV was calculated using the equation of Oser [31]: BV = ((1.09 × *EAAI*) − 11.70). The Protein Efficiency Ratio (PER) estimates the protein nutritional quality based on the amino acid profile after hydrolysis. PER was determined using the model developed by Ihekoronye [32]: PER = −0.468 + (0.454 × (Leucine)) − (0.105 × (Tyrosine)). The Nutritional Index (NI) normalizes the qualitative and quantitative variations of the test protein compared to its nutritional status. NI was calculated using the equation of Crisan and Sands [33], which considers all the factors with an equal importance:

NI = (*EAAI* × Protein (%)/100). (2)

### 2.8. Statistics

Data were subjected to analysis of variance using IBM SPSS Statistics 21 (IBM Corporation, New York, NY, USA), and significant differences (*p* < 0.05) between individual means were identified by Tukey’s test.

## 3. Results and Discussion

### 3.1. Chemical and Physical Characterisation of Fermented and Native Faba Flour

Fermentation did not have a significant impact on the protein (35% dm) and soluble starch (42% dm) contents of the faba flour, whereas total dietary fibre (DF) (7% versus 6.7% dm) and fat contents (1.5% versus 0.9% dm) were slightly but significantly reduced. Reduction in total DF and fat content by fermentation of various pulses such as pigeon pea, gram, kidney bean and cowpea has been observed earlier [34,35]. DF solubilisation is known to take place in fermentation of cereals largely due to the action of endogenous enzymes present in the raw material [36]. The dietary fibre content of faba bean is approximately 10% and consists mainly of insoluble fibre such as hemicelluloses [37]. The reduction in DF content might be also associated with the extensive depolymerisation resulting in hydrolysis products, which will not be analysed as DF, or on the ability of fermentative microorganisms to consume hydrolysis products [38].

Fermentation of faba bean flour reduced setback (native flour: 959 ± 13 cP, fermented faba bean flour: 169 ± 8 cP) and final viscosities (native flour: 1642 ± 8 cP, fermented faba bean flour: 864 ± 11 cP) in the RVA analysis of flour slurry, as reported earlier for various starch matrices including, for example, mung bean [39]. Setback viscosity is an indicator of starch retrogradation and can be reduced by decreasing the pH of the flour matrix as the starch granules become fragile and break down easily at low pH by heating [40]. The setback value for faba bean flour reduced by 83% after lactic acid fermentation, where the pH dropped from the initial value of 6.6 to 4.1. This might have lowered the degree of starch retrogradation after heating. Acidic treatment affects the pasting properties of starches by partial hydrolysis of glycosidic linkages of starch molecules [40]. The impact of fermentation on the other RVA parameters of faba flour such as peak and breakdown viscosity and pasting temperature were not significant.

### 3.2. Macro and Micro Structure of Bread

The impact of flour pre-fermentation on the structure of breads was evaluated both at macro- and micro-scales. Faba breads had higher volume than soy bread, whereas fermentation reduced the volume yield of bread. The volume yields were significantly different among the breads: 2.4 ± 0.1 ^a^ and 2.1 ± 0.1 ^b^ (mL/g) for unfermented and fermented faba bread respectively, and 1.5 ± 0.05 ^c^ mL/g for soy bread. GF bread dough generally resembles a cake batter and added proteins act as emulsifiers by forming a film or skin around oil droplets, which prevents structural failures due to coalescence. The good emulsifying capacity of faba bean proteins was thus reduced by fermentation [41]. Fermentation has been shown to reduce the foaming stability of faba bean flour by 66% [42], which at least partly explains to reduced functionality in gluten-free bread as well. The lowest volume, however, was for the soy bread (S37) used as a benchmark. Earlier, supplementation of gluten-free corn and potato starch-based breads with 10% soy protein concentrate (72% protein content, 7% protein in bread) decreased the specific volume [43]. In addition, supplementation of gluten-free rice flour-based bread with 13% to 25% (flour weight basis) of soybean protein isolate (81% protein content, 10.5%–20% protein in bread) had a detrimental effect on crumb structure [44]. Faba flour thus could be a better option for protein enrichment of GF bread.

**Figure 1:**
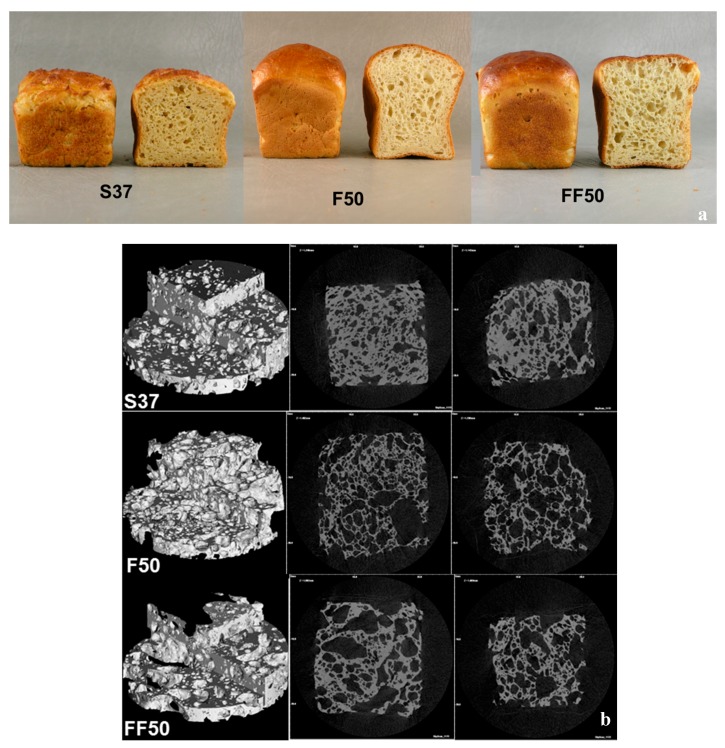
(**a**) Digital images of soy bread (S37), faba bread (F50), fermented faba bread (FF50). (**b**) Rendered three-dimensional (3D) images and selected 2D reconstructed X-ray microtomography (XMT) images of bread samples.

Faba breads had better loaf shape, smooth crust and better crumb colour and structure compared to soy bread (Figure 1a). The microstructural characterization results were in line with the macrostructural data as faba breads also had a more porous, open structure (Figure 1b, Table 1). Native and fermented faba breads (F50 and FF50) with the same 16% protein content as the soy bread (S37) had 82% and 72% total porosity respectively (Table 1), flour fermentation thus decreasing porosity. Faba bread had an air cell diameter of 690 µm, fermented faba bread of 561 µm, and soy bread of 230 µm, having the lowest value. The reduction in air cell diameter by fermentation of faba flour was not significant. On the other hand, fermented faba bread had the thickest cell wall (109 µm). We hypothesize that the cell wall thickness was mainly affected by the surface and functional properties of the faba bean protein. Furthermore, the hydrolysis of protein and reduction in foam formation and emulsification capacity of faba bean proteins by fermentation might have adversely affected the air cell formation during bread processing and resulted in smaller but thicker cell wall formation.

**Figure 2:**
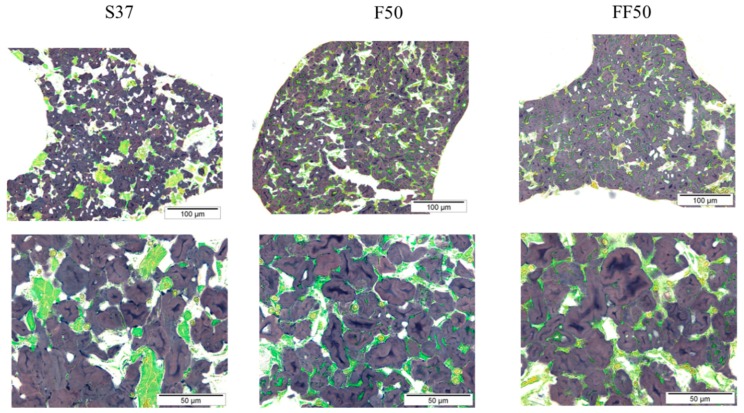
Light green and lugol’s iodine staining images of soy bread with 16% protein content (**S37**), faba bread with 16% protein content (**F50**), fermented faba bread with 16% protein content (**FF50**). Green represents protein and dark blue is starch (mainly amylose).

It has earlier been shown that fermentation improves the structure, texture and shelf life of wheat bread [45]. The literature is not, however, consistent for gluten-free baking. For example, in another plant protein-rich GF-bread matrix, replacing 20% of oat flour with oat sourdough (produced with *L. plantarum*) did not improve the loaf volume, aroma or microbiological shelf life of the gluten-free oat bread [46]. Lactic acid fermentation of soy-, rice- and buckwheat-based gluten-free mixture hydrolysed soy proteins after 24 h fermentation, but the impact of fermentation in improving texture and structure was minor compared to wheat baking [47]. The microstructures of bread samples as analysed using light microscopy are presented in Figure 2. As expected, no orientated or organized protein network typical for wheat breads was observed. In addition, starch was the continuous phase and protein appeared as the discontinuous phase. However, it is known that gelling properties of faba bean protein is better than soy protein, and the minimum concentration required for a protein dispersion to form a self-supporting network is 14% and 16% for faba and soy proteins, respectively [48]. This might have further contributed to the superior structural properties of faba breads compared to the control soy bread. Large soy protein aggregates (green) surrounded by starch (dark blue) was visible for soy bread (Figure 2). The protein aggregates in both faba breads were clearly smaller than in soy bread. For unfermented faba bread (F50), larger protein particles were dispersed throughout the continuous starch phase. After fermentation of faba flour, these protein particles are degraded to finer particles, as seen in fermented faba bread (FF50) (Figure 2).

### 3.3. Textural Properties 

**Figure 3:**
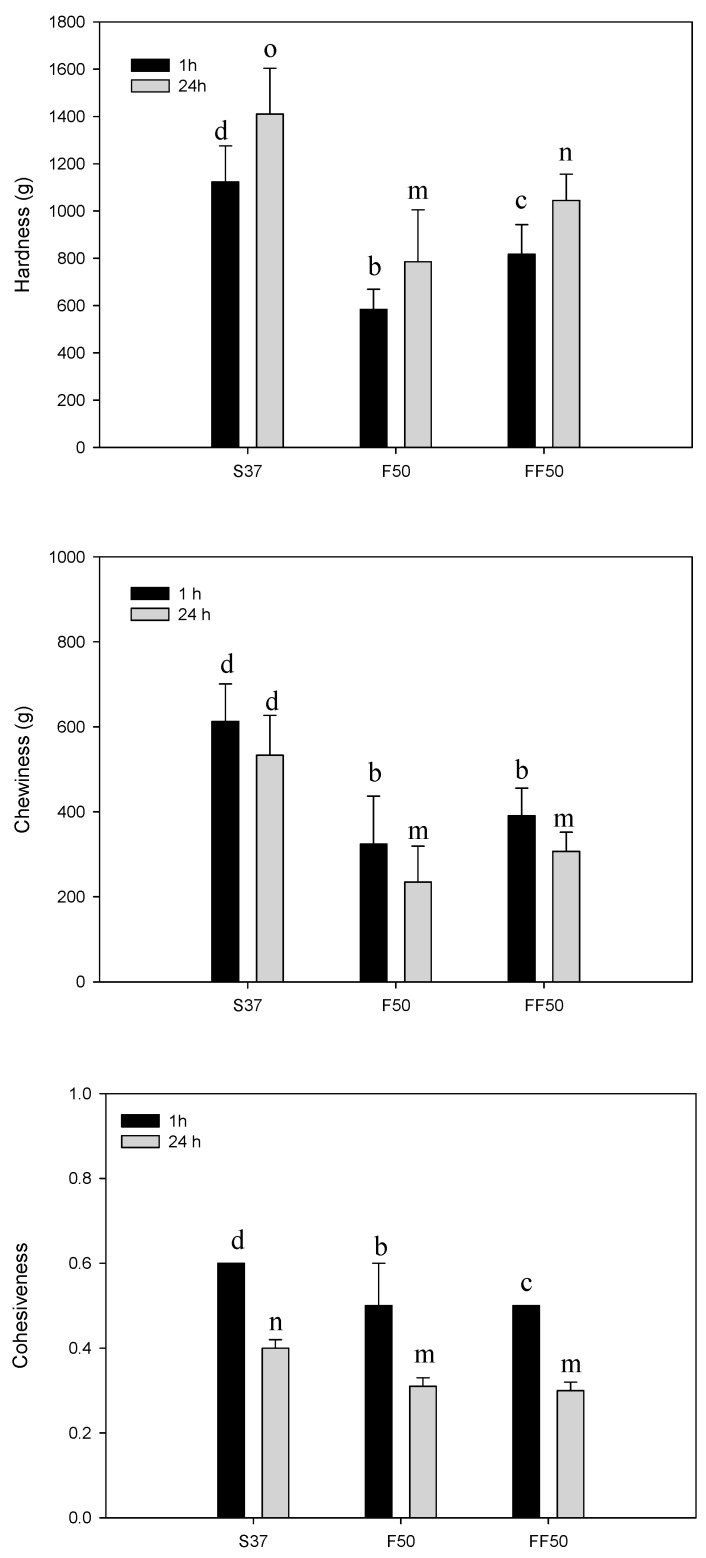
Textural parameters of bread samples after 1 h and 24 h of baking.

Hardness, chewiness and cohesiveness of the breadcrumb as a function of fermentation-induced changes and storage time are presented in Figure 3. Fermentation of faba flour prior to bread making significantly increased crumb hardness (F50 (584 g) versus FF50 (817 g). Air cell diameter, air cell wall thickness and porosity influence the textural properties of baked products [22]. Hardness was negatively related to bread volume, total porosity and pore size. Negative correlation between crumb hardness and volume has also been reported by others [20]. In the current study, the changes in protein structure were proposed to be the key factor responsible for the structural and textural differences as the amount of other structuring agents such as gums, emulsifiers and yeast was constant. Beyond 15% (flour basis) soy flour addition resulted in a hard, compact and low loaf volume for GF bread based on rice and cassava flour [49], whereas soy flour substitution levels lower than 12.5% gave structural failure (e.g., internal cracks). Faba bean protein has water holding capacity similar to soy protein but superior whippability and foam stability [50] which might explain better structural and textural properties of faba breads over soy bread. However, solubility of faba bean protein is reduced at pH levels 4–5. After 48 h of fermentation, the pH of faba flour was 4.1 but before freeze drying it was adjusted to higher pH levels around 5.5 by using sodiumbicarbonate.

Earlier, freeze drying of faba bean protein isolates was shown to reduce the solubility and emulsification properties in protein/water suspension with a maximum 4% protein content compared to spray-dried counter parts [51]. The effect was stated to be lower when protein content increased [51]. As the protein content in this study is much higher (16% of flour mix), the effect of freeze-drying on protein functionality can be expected to be minor compared to the effects caused by fermentation. The TTA values of bread doughs after proofing was 4.66 ± 0.16, 5.53 ± 0.05, 11.04 ± 0.15 mL of NaOH 0.1 M/10 g of dough for S37, F50 and FF50 respectively, whereas the pH values were 6.5, 6.6 and 5.6.

Degradation of the protein matrix by acidification and weakening of the structure reduced the carbon dioxide holding capacity of dough, lowering both the bread volume and porosity that further increased hardness. For all breads, hardness value significantly increased the next day. Chewiness of all faba breads was lower than soy bread (Figure 3). Chewiness reduced after 24 h storage for all breads despite the increase in hardness, which could be linked to the pronounced reduction in cohesiveness. Faba breads were less cohesive than soy bread (Figure 3). Soy crumb was wet and had a more plastic structure (Figure 1a), which could explain the higher cohesiveness value. Fermentation slightly but significantly reduced the cohesiveness of faba breads when they were fresh, but no significant difference was observed after 24 h storage.

### 3.4. Sensory Properties

The general appearance of faba breads was better than soy bread in terms of evenness, shape and colour intensity of bread (Table 2). No significant difference was caused by fermentation. Crumbliness, evenness of pore size and springiness of crumb were similar for all breads. However, F50 bread made with unfermented faba flour had the highest score for crumb softness. Intensity of colour was similar for all breads (Table 2), whereas fermentation of the faba flour prior to bread making increased the intensity of crumb flavour. During lactic acid fermentation proteolysis, acidification and formation of volatile compounds influenced the flavour profile [21].

### 3.5. Nutritional Characteristics 

Bread with fermented faba bean flour (FF50) had a significantly lower hydrolysis index (76 ± 1 ^c^) compared to S37 soy bread (93 ± 4 ^b^). However, there was no significant difference of HI values between FF50 and F50 (83 ± 2 ^bc^). Processing can induce macromolecular interactions between starch and components of the food matrix reducing the availability of starch for digestive enzymes. The chemical changes and interactions between starch-protein that occur in the presence of lactic acid have been suggested to lower the rate of starch hydrolysis [13]. Lactic acid fermentation reduced the in vitro starch hydrolysis rate for sorghum and teff sourdough bread whereas it increased for quinoa and buckwheat sourdough breads [46,52]. The authors attributed the varying effect of fermentation to the structural factors such as increased loaf volume and higher porosity in quinoa and buckwheat sourdough breads [46,52].

Proteins are key components, which contribute to the nutritional value of foods. The quality of proteins is estimated through their amino acid composition, which, combined with protein digestibility, may predict the nutritional value. In vitro digestibility gives information about the stability of protein hydrolyzates, and resistance to digestion. Fermentation increased the in vitro protein digestibility of breads from 53.9% to 72.3% (Table 3) to values even higher than that of soy bread (64.8%) (Table 3). This might be attributed to proteolysis during LAB fermentation [28]. Furthermore, fermentation increased almost all the chemical scores of faba bread to values higher than those of soy bread. All samples contained essential amino acids and the chemical score indicated that Methionine and Cysteine were, respectively, the first and second limiting amino acids. EAAI indicates the ratio of essential amino acids of the sample compared to the reference. BV estimates the nitrogen potentially retained by the human body after consumption. Both EAA and BV indexes were significantly higher for fermented faba bread (FF50) compared to unfermented faba bread F50 and soy bread (S37). The Protein Efficiency Ratio (PER), which reflects the capacity of a protein to support the body weight gain, increased by fermentation from 32.7 to 35.7 but was still lower than soy bread (37.1). Among the indexes that are used to evaluate the nutritional value of foods, only the NI combines qualitative and quantitative factors. Indeed, NI is considered a global predictor of the protein quality. The Nutritional Index (NI) of faba bread (F50, 1.57) increased by fermentation (FF50, 2.47) to levels even higher than the soy bread (S37, 2.1). Fermentation markedly increased protein bioavailability.

## 4. Conclusions

The present study showed that fermentation can be used as a modification tool particularly for improving the nutritional properties of faba bean flour. Fermentation of faba bean flour increased in vitro protein digestibility (72.3% versus 53.9%) of gluten-free faba breads. EAA and BV indexes as well as PER were significantly increased for breads made with fermented faba bean flour. Furthermore, fermentation increased the NI of faba bread (2.47) to levels even higher than the soy bread (2.1). No significant difference was caused by fermentation on the general appearance of faba breads (evenness, shape and colour intensity). A sensory panel indicated that crumbliness, evenness of pore size and springiness of crumb were similar for all breads.

Generally, gluten-free products are inferior in their nutritional properties as they are mainly high in carbohydrates and low in protein and dietary fibre. The incorporation of fermented faba bean flour will not only improve nutritional quality, as stated above, but also enable a source of protein (a minimum of 12% of the energy value of the food is provided by protein) or high-protein (a minimum of 20% of the energy value of the food is provided by protein) claims in gluten-free products if used as a protein ingredient. The gluten-free faba bean bread of this study would be qualified for “source of protein” and “source of fibre (at least 3 g of fibre per 100 g of product)” claims.

## Figures and Tables

**Table 1:** Microstructural parameters of image analysis data by XMT.

Bread Samples	Average Cell Wall Thickness (µm)	Average Cell Diameter (µm)	Total Porosity (%)
S37	63 ± 9 ^d^	230 ± 54 ^a^	61 ± 2 ^a^
F50	87 ± 11 ^e^	690 ± 8 ^bc^	82 ± 2 ^c^
FF50	109 ± 29 ^f^	561 ± 61 ^b^	72 ± 4 ^b^

Means in the same column with different letters are significantly different (*p* < 0.05).

**Table 2:** Sensory properties of bread samples (1 h after baking).

	S37	F50	FF50
Appearance			
Evenness of bread	2.2 ± 0.7 ^a^	3.6 ± 0.6 ^b^	3.5 ± 0.3 ^b^
Shape of bread	2.4 ± 0.9 ^a^	3.7 ± 0.5 ^b^	3.8 ± 0.5 ^b^
Intensity of bread colour	2.5 ± 0.4 ^a^	3.5 ± 0.2 ^b^	4.0 ± 0.3 ^b^
Texture of the crumb			
Crumbliness	2.0 ± 0.1 ^a^	2.3 ± 0.4 ^a^	1.9 ± 0.4 ^a^
Evenness of the pore size	3.5 ± 0.2 ^a^	3.3 ±0.3 ^a^	3.3 ± 0.2 ^a^
Softness of the crumb	3.3 ± 0.3 ^ab^	4.2 ± 0.2 ^c^	3.0 ± 0.8 ^a^
Springiness of the crumb	3.7 ± 0.7 ^a^	3.9 ± 0.4 ^a^	3.7 ± 0.5 ^a^
Toughness	1.7 ± 0.3 ^a^	1.3 ± 0.3 ^a^	1.7 ± 0.7 ^a^
Crumb flavour and colour			
Intensity of the colour	2.8 ± 0.5 ^ab^	2.5 ± 0.7 ^a^	3.5 ± 0.7 ^ab^
Intensity of the flavour	2.8 ± 0.5 ^ab^	2.5 ± 0.8 ^a^	3.5 ± 0.7 ^ab^

Means in the same row with different letters are significantly different (*p* < 0.05).

**Table 3:** Nutritional indexes of breads.

	S37	F50	FF50
In vitro protein digestibility (%)	64.8 ± 0.1 ^c^	53.9 ± 0.2 ^d^	72.3 ± 0.2 ^b^
Chemical score (%)			
Histidine	88 ± 2 ^b^	85 ± 1 ^c^	92 ± 2 ^a^
Isoleucine	72 ± 2 ^a^	65 ± 2 ^b^	64 ± 2 ^b^
Leucine	96 ± 1 ^a^	88 ± 2 ^b^	96 ± 2 ^a^
Lysine	105 ± 2 ^b^	113 ± 3 ^a^	114 ± 1 ^a^
Cysteine	35 ± 2 ^c^	33 ± 1 ^c^	51 ± 2 ^a^
Methionine	27 ± 1 ^c^	29 ± 1 ^c^	32 ± 1 ^b^
Phenylalanine + Tyrosine	58 ± 1 ^b^	49 ± 1 ^c^	63 ± 2 ^a^
Threonine	74 ± 3 ^b^	78 ± 2 ^a^	78 ± 1 ^a^
Valine	70 ± 2 ^a^	69 ± 2 ^a^	70 ± 2 ^a^
Tryptophan	35 ± 1 ^c^	44 ± 1 ^b^	62 ± 2 ^a^
Sequence of limiting EAA			
	Methionine	Methionine	Methionine
	Cysteine	Cysteine	Cysteine
	Tryptophan	Tryptophan	Tryptophan
Protein score (%)	27 ± 2 ^c^	29 ± 1 ^c^	32 ± 2 ^b^
Essential Amino Acid Index (EAAI)	61.5 ± 0.4 ^b^	58.4 ± 0.3 ^c^	63.4 ± 0.4 ^a^
Biological Value (BV)	55.4 ± 0.3 ^b^	51.9 ± 0.1 ^c^	57.4 ± 0.4 ^a^
Protein Efficiency Ratio (PER)	37.1 ± 0.4 ^a^	32.7 ± 0.3 ^c^	35.7 ± 0.4 ^b^
Nutritional Index (NI)	2.1 ± 0.07 ^c^	1.57 ± 0.12 ^d^	2.47 ± 0.08 ^b^

Data are expressed as the mean of the results collected in two independent baking tests. ^a–d^ Values in the same row with different superscript letters differ significantly (*p* < 0.05).
